# Pathological Mechanisms Underlying Myalgic Encephalomyelitis/Chronic Fatigue Syndrome

**DOI:** 10.3390/diagnostics9030080

**Published:** 2019-07-20

**Authors:** Daniel Missailidis, Sarah J. Annesley, Paul R. Fisher

**Affiliations:** Department of Physiology Anatomy and Microbiology, La Trobe University, VIC 3086, Australia

**Keywords:** myalgic encephalomyelitis, chronic fatigue syndrome, ME/CFS, diagnosis, metabolism, mitochondria, inflammation, immune system, signaling, gut microbiota

## Abstract

The underlying molecular basis of myalgic encephalomyelitis/chronic fatigue syndrome (ME/CFS) is not well understood. Characterized by chronic, unexplained fatigue, a disabling payback following exertion (“post-exertional malaise”), and variably presenting multi-system symptoms, ME/CFS is a complex disease, which demands a concerted biomedical investigation from disparate fields of expertise. ME/CFS research and patient treatment have been challenged by the lack of diagnostic biomarkers and finding these is a prominent direction of current work. Despite these challenges, modern research demonstrates a tangible biomedical basis for the disorder across many body systems. This evidence is mostly comprised of disturbances to immunological and inflammatory pathways, autonomic and neurological dysfunction, abnormalities in muscle and mitochondrial function, shifts in metabolism, and gut physiology or gut microbiota disturbances. It is possible that these threads are together entangled as parts of an underlying molecular pathology reflecting a far-reaching homeostatic shift. Due to the variability of non-overlapping symptom presentation or precipitating events, such as infection or other bodily stresses, the initiation of body-wide pathological cascades with similar outcomes stemming from different causes may be implicated in the condition. Patient stratification to account for this heterogeneity is therefore one important consideration during exploration of potential diagnostic developments.

## 1. Introduction

Myalgic encephalomyelitis/chronic fatigue syndrome (ME/CFS) encompasses diverse symptoms that manifest variably across a range of body systems, the characteristic symptoms being chronic unexplained fatigue (lasting more than 6 months) and post-exertional malaise (PEM)—a disabling and exacerbated disease state following bouts of physical or mental exertion that exceed a patient-specific threshold. ME/CFS also entails a varied kaleidoscope of other symptoms including muscle weakness, migraine, flu-like symptoms, cognitive impairment (“brain fog”), and sensitivities to a variety of external stimuli that may include light, sound, or specific odors. This can be accompanied by comorbidities, such as fibromyalgia, postural orthostatic tachycardia (POTS), and Ehlers–Danlos syndrome. 

A major challenge for this field of study has been the varied usage of multiple diagnostic case criteria that may render comparison between studies difficult depending on the composition of the participant cohort. Furthermore, these criteria are slow processes of exclusion that leave patients without the support or acknowledgment that they need for extended periods and they may be subjected to a protracted, harsh, and insensitive diagnostic gauntlet. These problems are further compounded by medical guidelines in some developed countries that are out of date regarding ME/CFS clinical practice and require urgent overdue revision. 

Case definitions, such as the commonly termed Oxford [1] or Fukuda [2] criteria, are most often utilized throughout the UK and USA, respectively, yet may fail to discriminate between generalized chronic fatigue and ME/CFS which specifically also involves PEM, which aids in characterizing this disorder as a discrete clinical entity. Also in usage are the Canadian Consensus Criteria [3] and International Consensus Criteria [4], which mandate PEM for a diagnosis of ME/CFS and therefore may be considered more specific definitions. While the presence of PEM is an optional component of the Fukuda criteria, PEM is, unfortunately, not required for research participation by all studies using this or other less strict definitions. Consequently, the discovery of a reliable diagnostic biomarker is perhaps the most common recurring theme in modern ME/CFS research. Despite myriad relevant study outcomes [5,6,7,8,9,10,11,12,13,14,15,16], no such discovery has yet been widely validated or implemented as a suitable diagnostic biomarker of ME/CFS.

Not only does ME/CFS affect multiple body systems and organs, but it does so with different and time-varying levels of severity and different patterns of comorbidities in different individuals, thereby producing a highly heterogeneous patient population [7,17,18,19,20,21,22,23]. This complexity represents a major challenge to the task of incriminating one underlying pathological mechanism. It is also possible that different causative molecular insults result in different subsequent clinical presentations and this could contribute towards heterogeneity in the disorder. Patient subtyping to manage this heterogeneity has been previously discussed in the field [20,23] and is lent credence by reproduced patterns of differential disease-associated gene expression [24,25,26], gene expression profiles concurrent with comorbid POTS [27], distinct DNA methylation profiles associated with quality of life scores and PEM [28], severity and frequency of physical or mental fatigue [29], or irritable bowel syndrome (IBS) comorbidity [30], which can be concurrent with specific changes to patient metabolism [31]. As timely, objective, and accurate diagnosis remains the most clear challenge facing the field, patient subtyping may be an important component of new diagnostic techniques and has seen early investigation with stratification-based severity scores [32] or cytokine co-expression patterns [33]. 

In summation, ME/CFS etiology has been difficult to pin down due to the combination of a diagnostic quagmire and the disorder’s heterogeneous symptom presentation across multiple body systems. A traditional view has held that ME/CFS onset is often precipitated by some manner of bodily insult, commonly infection, however, the disorder is left without any known single causative pathogen to date. Given the heterogeneity of the patient population, this is unsurprising. It is possible that the initial pathological insult may not always be pathogen-mediated and could instead be instigated by alternative stresses of sufficient magnitude as to nudge homeostatic regulation loops into alternative stable states [34], and these varying modes of initial insult may contribute to the heterogeneity of ME/CFS clinical presentation. 

Current research shows a tangible biomedical foundation underlying this clinical puzzle. Most of this evidence pertains to disturbed muscle function, metabolism, mitochondria, immunity, signaling, neurological, adrenal, and gut health. It is possible that these threads are together entangled as parts of an underlying molecular pathology reflecting a far-reaching homeostatic shift influencing each of these systems, perhaps differentially between individuals with varying clinical features. Furthermore, evidence of abnormalities affecting multiple systems is based on associations and the causal mechanisms responsible for the underlying pathology have yet to be determined. The following sections will address the current evidence for dysfunction across these systems in ME/CFS with specific examples of potential pathological interactions. 

## 2. Abnormal Metabolism and Mitochondrial Function

The nature of the persistent fatigue and PEM experienced by patients renders the area of cellular energetics fertile ground for investigation. However, in the intervening years since early studies [35,36,37], mitochondria had been largely neglected in the field until their re-emergence as an area of interest during the last 10 years [9]. The recent interest in this area has since generated a basis to support some manner of both mitochondrial and broader metabolic dysfunction in ME/CFS. 

### 2.1. Dysregulated Amino Acid Metabolism and Impaired Provision of TCA Cycle Substrate

This accumulation of evidence supporting dysregulated metabolism and mitochondrial energetics in ME/CFS has taken place across many experimental areas. There have been recent studies utilizing the metabolomics approach, which captures a quantitative snapshot of steady-state metabolite levels in a sample to infer underlying biochemical pathway modulation, typically achieved by either mass spectrometry (MS) or nuclear magnetic resonance (NMR) spectroscopy applications. The first of these studies used NMR to interrogate analytes within ME/CFS blood samples and reported decreases in glutamine and ornithine concentrations, suggesting abnormal amino acid metabolism linked to urea cycle dysregulation [38]. A subsequent study utilizing MS conversely reported an elevation in ornithine concentration with a decrease in citrulline, but this also suggests urea cycle dysregulation [6]. 

Subsequent work undertaken by the authors of the first study proposed that impaired glycolytic formation of pyruvate could be providing less downstream oxidized pyruvate derivatives to be used as substrate for the tricarboxylic acid (TCA) cycle [39]. Work by others suggested that instead of a reduction in the glycolytic pyruvate supply, a deficiency in pyruvate dehydrogenase (PDH) function may form a bottleneck for the provision of TCA cycle substrate [40]. These and the previous amino acid discrepancies could be due to differences in techniques (NMR vs. MS) and thus the range of detectable molecules. What could be taken from both lines of approach—supported by data from other similar studies [6,13,31] and one cell culture study [41]—is that there may be some manner of TCA cycle disturbance in ME/CFS, possibly one that is substrate supply-driven. 

### 2.2. Inefficient ATP Synthesis and Abnormal Energy Stress Signaling

If the TCA cycle output of oxidative phosphorylation (OXPHOS) complex substrates is reduced by such a glycolysis [39] or PDH [40] defect, one might expect disturbances in cellular energy production in ME/CFS cells. There have been several studies which report a reduction of steady-state ATP levels [9,42,43], yet other studies have reported an elevation [44]. However, these steady-state measures do not provide information as to the metabolic flux (rates of production and depletion) of the molecule of interest. Real-time parameters of aerobic respiration and glycolysis can be measured in live cells by extracellular flux assays, which measure oxygen consumption rates (OCRs) and extracellular acidification rates (ECARs) using intact cells. Published studies of this type both found no significant difference in absolute ATP synthesis rates between the ME/CFS and control cells [41,45]. However, this does not necessarily mean that there is no ATP synthesis defect, since a defect/inefficiency in ATP synthesis could be offset by compensatory homeostatic mechanisms.

The homeostatic regulation of cellular energy metabolism is centered on two stress-sensing protein kinases, AMP-activated protein kinase (AMPK) [46] and target of rapamycin (TOR) [47], which play key, often mutually inhibitory, roles. If their activities are chronically dysregulated by metabolic abnormalities and energy insufficiencies in ME/CFS cells, they may be unable to respond to additional energy demand. This is supported by reports of AMPK in muscle cells from people with ME/CFS being unresponsive to stimulation by contraction-induced ATP depletion [43,48]. Such insensitivity could result from AMPK already being in an activated state in these cells, or to its inhibition by chronically hyperactivated TOR complex 1 (TORC1). Elevated TORC1 activity was recently observed in ME/CFS cells (lymphoblasts) and this was accompanied by inefficient mitochondrial ATP synthesis and abnormally high and presumably compensatory expression of mitochondrial proteins [41]. Elevated expression of mitochondrial proteins has also been found in other studies of patient saliva, lymphocytes, and platelets [41,49,50,51]. Furthermore, reduced creatine kinase (CK) levels in the serum of people with ME/CFS may suggest reduced cellular CK presence [15], which could contribute to inefficient ATP synthesis given the enzyme’s roles in ATP homeostasis [52]. Despite the breadth of sample types tested that suggest dysregulated energy metabolism (serum, urine, feces, muscle, B cells, lymphoblasts), it remains to be directly demonstrated that the perturbation of the associated stress-sensing pathways is systemic and this should be an area of future investigation. 

As noted above, glycolytic catabolism of glucose is a major supplier of acetyl coenzyme A (CoA) to the TCA cycle, and this can be assayed in intact cells by measuring the rate of acidification of the medium by cells provided with glucose as a substrate. This has been reported recently using natural killer (NK) cells from a small sample of six patients and six healthy controls [53]. Although the authors found no differences in aerobic respiration rates, they did observe a reduced glycolytic reserve in the ME/CFS cells. The glycolytic reserve is a measure of the excess capacity of glycolysis to meet cellular ATP demands when mitochondrial ATP synthesis by oxidative phosphorylation is inhibited. This small study reported individual O_2_ consumption and extracellular acidification rates that were either negative or very small positive values, placing them at the threshold of reliable detection. This caveat also applies to heterogeneous peripheral blood mononuclear cell (PBMC) populations, which are commonly used for extracellular flux respirometry and have been previously employed in ME/CFS work [45]. This difficulty arises because peripheral blood lymphocytes are metabolically quiescent [54] and this not only makes metabolic rate assays technically difficult, but it may obscure differences in metabolism that would be apparent in actively metabolizing cells. 

### 2.3. A Shift Towards Lipid Metabolism

The TCA cycle can also be provided with acetyl CoA produced by fatty acid beta-oxidation when glucose-derived sources are insufficient [55]. If the provision of substrate to the TCA cycle is indeed deficient in ME/CFS as previously suggested [31,39,40], a role for the compensatory elevation of fatty acid metabolism could be implicated in the disorder [41]. Fatty acid synthesis and beta-oxidation are regulated by both AMPK [56] and TORC1 [57]. AMPK promotes fatty acid beta-oxidation when activated by elevated ATP demand and promotes fatty acid biosynthesis when inactive, while TORC1 exerts the opposite effects. AMPK and TORC1 regulate each other in a complex reciprocal feedback network [58], so that it is also possible for scenarios to arise where both are simultaneously activated [59]. This raises the question: If both AMPK [43,48] and TORC1 [41] activities are elevated in ME/CFS cells, would we expect the rates of fatty acid catabolism to be increased or decreased? An answer may be found within the specific mechanisms of regulatory action of these pathways in lipid homeostasis. Activation of TORC1 promotes fatty acid biosynthesis by elevating the expression of gene products including acetyl-CoA carboxylase (ACC) through the upregulation of transcription factors Sterol Regulatory Element Binding Proteins 1 and 2 (SREBP-1 and SREBP-2) [60]. ACC activity results in an accumulation of malonyl CoA, which is a potent inhibitor of the mitochondrial import of fatty acids for beta oxidation. ACC, however, is a primary regulatory target of AMPK and is inactivated by phosphorylation when AMPK is activated [61]. In this way, the concurrent activation of AMPK and TORC1, if it does indeed occur in ME/CFS cells, may allow AMPK to constrain the effects of TORC1’s upregulation of lipid-biosynthesis and downregulation of beta-oxidation. At the same time, both AMPK and TORC1 directly or indirectly induce the expression of diverse mitochondrial proteins, including those involved in fatty acid beta oxidation. The combined effects could be a steady state in which the cells have increased their use of beta oxidation relative to glycolysis as a supplier of acetyl CoA to the TCA cycle. Fatty acid oxidation is normally upregulated as a supplementary energy pathway during fasting or exercise as a response to reduced blood glucose concentration [62]. An increased reliance on fatty acid oxidation, even at rest, may therefore also contribute to the inability for people with ME/CFS to meet the elevated energy demands imposed by exertion. Such a switch to lipid oxidation can be mediated by elevated inflammation [63] and in combination with the pathological inflammation seen in patients [64] could be evidence of a pathological interaction between inflammation and metabolism in ME/CFS. 

## 3. Disturbed Immunity, Signaling, and Inflammatory Pathways

### 3.1. NK Cells

The function of the immune system has been a focus of ME/CFS research for many years. Evidence for immune dysfunction in ME/CFS has largely been sought through study of NK cells, which are cytotoxic immune cells with roles in both the innate and adaptive immune responses. Multiple groups have reported reduced NK cell cytotoxicity or numbers [65,66,67,68,69] combined with concordant alterations to functional surface markers [70,71,72]. Conversely, other groups have reported increased cytotoxicity in combination with alterations to functional surface markers. For example, perforin, a glycoprotein used as a functional indicator of NK cell cytotoxicity due to its roles in NK cell mediated lysis [73,74], has been reported as upregulated [71], downregulated [65], or, along with every other assessed phenotypic parameter, unaltered in people with ME/CFS [75]. A recent, rigorous large-scale biobank study also found no significant differences in NK cell numbers, subtype composition, or assessed functional parameters [76]. In summation, NK research remains an area of active interest, but in light of conflicting findings, the role of NK cells in the disorder is still not well understood. 

### 3.2. Calcium Signaling

Calcium signaling is crucial to immune cell function [77] and is tied to the mitochondria and endoplasmic reticulum as hubs of regulatory control and calcium storage [78]. Therefore, a disturbance to calcium signaling could contribute to pathological outcomes involving immune system or bioenergetic dysfunction, both of which have been implicated in ME/CFS. In addition to the previous evidence of altered surface proteins on NK cells from ME/CFS patients, a reduction in the expression of transient receptor potential melastatin 3 (TRPM3) calcium ion channels [79] has been reported in a subpopulation of ME/CFS NK cells [80,81,82]. The reason for the reduced expression of TRPM3 in these cells is unknown, but in other cell types, expression of TRPM3 is repressed by the activity of microRNA-204 (miR-204), encoded by intron 6 of the *TRPM3* gene [83]. However, miR-204 is not amongst the microRNAs whose expression is reportedly altered in ME/CFS patients [84]. 

Reduced expression of TRPM3 receptors would be expected to cause a reduction in Ca^2+^ responses to pregnenolone sulfate (PregS), a specific activating ligand for TRPM3 channels. However, the opposite was observed when Ca^2+^ levels were assayed by flow cytometry using Indo1, a Ca^2+^-sensitive fluorescent dye in the TRPM3-depleted NK cells [80]. Subsequent studies using whole cell patch clamping, however, have reported a loss of PregS-stimulated Ca^2+^ responses [81,82]. 

### 3.3. Links to Mitochondrial Dysfunction

Cytosolic Ca^2+^ is a key element in the cell’s extensive homeostatic networks, such that Ca^2+^ signals are regulated by and play regulatory roles in multiple processes in cells, including mitochondrial respiratory activity [85] as well as mitochondrial protein import [86] and the ion transport activity of TRPM3 itself [87], both of which are activated by Ca^2+^-calmodulin. The mitochondria serve as both a source and a sink for cytosolic Ca^2+^ and the mitochondria interact closely with the endoplasmic reticulum at sites that mediate the exchange of lipids and Ca^2+^ signals [88]. The mitochondria thus form a hub of interaction between Ca^2+^ and other molecular signals that regulate mitochondrial function, such as AMPK and TORC1 activity. In NK cells specifically, where the calcium-signaling defect has been reported, TORC1 activity is required for both effector function and cytokine production [89,90,91] and it has been suggested that the development of NK cell antiviral memory is influenced by mitochondrial function [92]. The reports of altered NK effector function in ME/CFS could therefore also be related to abnormal mitochondrial function and TOR signaling. Disrupted intracellular signaling and mitochondrial function in ME/CFS have also been previously linked through myriad other pathways provoked by immune-inflammation and oxidative stress [93]. 

### 3.4. Inflammation

Chronic system-wide inflammation is thought to be central to ME/CFS in the clinical setting as it is associated with symptom severity [64], but the evidence demonstrating a role for specific pro-inflammatory cytokines is inconsistent. While there are indeed reports of the elevation of various pro-inflammatory cytokines in ME/CFS [33,71,94,95,96,97,98,99], which would tie in with the chronic inflammation in the clinical setting, these findings contrast with reports of reduced expression of pro-inflammatory agents, such as interleukin-8 or transforming growth factor-beta1 [100,101]. Consequently, evidence for the specific directional shift of individual cytokines has been previously summarized as largely inconsistent [102]. Despite these issues, cytokine expression has been previously associated with ME/CFS disease duration and may have value in aiding the stratification of patient cohorts [33,94]. One group has shown that a cohort of ME/CFS patients with leaky gut syndrome as a comorbidity may undergo significant symptom remission when the IgM and IgA immune responses are attenuated by treatment with anti-inflammatory and antioxidant medications [103,104]. This finding has contributed towards the potential link between gut hyperpermeability and inflammation in ME/CFS. Therefore, it is likely that dysregulated chronic inflammatory action by the immune system is an aspect of ME/CFS, despite inconsistent reports.

### 3.5. Autoimmunity

Autoimmunity in the condition is an area that remains little researched, and has been most recently summarized and presented as a hypothetical model for ME/CFS, with some ties to gut dysbiosis and aberrant metabolism [105]. Other autoimmune models for ME/CFS have been previously based on rituximab’s role in B cell depletion as a possible therapeutic based on the promising outcomes of earlier studies [106,107]. This would be concordant with other reports of elevated naïve and transitional B cells in patients, which may suggest autoimmune tendencies [108]. However, a role for such B cell-mediated autoimmunity in the disorder is now challenged by the negative outcome of the more recent rituximab phase III clinical trial [109], which refutes the previous rituximab work. This may also indicate that autoimmunity only applies as a key pathomechanism in a small subset of patients who respond positively to rituximab treatment [110]. Other direct lines of evidence for autoimmune behavior in the disorder come from elevated autoantibody levels in sera [111], supported by the improvement of symptoms following autoantibody removal treatment [112], and abnormal IgM immune recognition of both microbial and human heat shock protein 60 in a subset of patients [113] or against phosphatidylinositol [114], despite the absence of an infective pathogen in each case. 

## 4. Implications of Altered Gut Microbiota and Physiology 

A disturbed gut microbiota [12,115,116,117,118,119,120,121] has been proposed to play a role in ME/CFS. This is accompanied by physiological gut abnormalities, such as impaired motility [122], elevated intestinal wall permeability [103,104,123,124], and IBS comorbidity [31,125,126], which has been proposed to comprise part of a ME/CFS subtype [30]. The implications of these disturbances could be far-reaching, since the intestinal microbiota both regulates [127,128] and is regulated by [129,130] the immune system. Additionally, many studies have demonstrated a link between the gut microbiota and host mitochondrial function or metabolism, with disturbances in one resulting in subsequent dysfunction in the other [131,132,133,134,135,136]. 

### 4.1. The Gut Microbiota and Metabolism

People with ME/CFS have been reported to present with gut microbiota disturbances and either metabolite variation [31,118] or mitochondrial dysfunction [116] and the two conditions could be linked. Sheedy et al. observed elevated Gram positive intestinal bacteria, which produce lactic acid that may lower the gut pH and lead to elevated gut permeability [137]. Furthermore, the translocation of these enteric lactic acid products into the bloodstream could contribute to the elevated lactate reported in the cerebrospinal fluid of ME/CFS patients [138,139,140] and in the blood of a subgroup of patients [141]. This contrasts with reports of reduced blood lactate as measured by H-NMR metabolomics [39], which suggests that lactic acidosis may only affect a subgroup of patients. If IBS comorbidity accompanied by gut dysbiosis and hyperpermeability is indeed a subtype of ME/CFS, the translocation of lactic acid produced by abnormally enriched Gram positive enteric bacteria in the affected individuals, rather than excessive production by the host metabolism, could explain this inconsistency. 

### 4.2. Pathological Interactions between Gut Hyperpermeability and the Mitochondria

Broader implications for the immune system and mitochondrial function may come from reports of increased translocation of immunogenic bacterial secretions from the gut into the bloodstream, which in ME/CFS may be mediated by intestinal wall hyperpermeability in patients affected with IBS [104,142]. For example, the generation of excess free radicals, which occurs in the mitochondria [143], has been proposed to form part of a microbial defense mechanism [144,145,146], with enteric species, such as *Escherichia coli*, highly susceptible to the bactericidal properties of free radical derived reactive nitrogen species (RNS) [147]. Excess free radical generation not only results in the formation of RNS but also reactive oxygen species (ROS), whose production may be elevated in ME/CFS given the many reports of elevated oxidative stress in the disorder [148,149,150,151,152,153]. Therefore, excessive free radical generation as such an antimicrobial response to circulating antagonists of enteric origin could provide one explanation for the reports of elevated oxidative stress in the disorder. Such a response may indeed be elicited by the ordinarily commensal Gram negative bacteria *E. coli*, which, while also known to target the mitochondria by the secretion of other toxins [154,155], secretes immunogenic lipopolysaccharide (LPS). This endotoxin secretion is normally suppressed by the host microbiota at large [131], however, the composition of the host microbiota is reportedly altered in cases of ME/CFS [12,115,116,117,118,119,120,121]. Such a disturbance to the normal LPS secretion-suppressing host microbiota in ME/CFS and the presence of gut hyperpermeability may therefore not only lead to the synergistic amplification of bacterial toxin translocation into the blood and the consequent activation of inflammatory pathways [104,142], but may also expose body-wide mitochondria to circulating virulent factors produced by Gram negative antagonists, such as *E.coli*. This is supported by the increase in *E. coli* gut colonization reported in cases of IBS [156], which, again, can be an ME/CFS comorbidity. 

### 4.3. The Gut–Brain Axis: Autonomic and Hormonal Dysregulation

Physiological stress, considered one predisposing factor for ME/CFS [157], has been suggested to play a role in modifying the gut microbiota in such a way that would reduce the numbers of *Bifidobacterium* and *Lactobacillus*, two genera responsible for the suppression of LPS-secreting commensal bacteria and has been implicated in this way in ME/CFS [158,159]. While the translocation of immunogenic LPS into the bloodstream is generally associated with body-wide inflammation [160,161], it may also lead to the elevation of proinflammatory elements and the stress hormone cortisol in the brain [162]. The transduction of immunogenic pathogen signals is but one aspect of the gut–brain axis, so it is pertinent to address the potential for broader gut-brain driven [163,164] or autonomic nervous dysregulation that may play a part in the disorder. Indeed, there is some evidence to support such dysregulation associated with vasomotor abnormalities [165] or mitochondrial Coenzyme Q10 deficiency associated with cardiovascular lesions [166]. There are relevant, respectively, to the ME/CFS comorbidity POTS, which likely involves vasomotor dysfunction [167], or to the cardinal symptom of chronic fatigue. Further, the linkage of cortisol-driven stress responses to the gut through the hypothalamic-pituitary-adrenal (HPA) axis [168] could be dysfunctional in ME/CFS due to the hypocortisolism previously suggested to play a role in the condition [169,170]. This possibility could weave another perturbed regulatory loop into the system, which may aid in perpetuating an altered homeostasis. This has been previously reviewed at length [171]. 

## 5. Multi-System Pathological Interactions

### 5.1. Exposure to Stressors and the Theoretical Homeostatic Perpetuation of a Disease State

The reported disturbances to ME/CFS metabolism have been linked to chronic activation of emergency cell survival mechanisms to cope with adverse conditions [7]. These adverse conditions could include energy stress, inflammation, HPA axis dysregulation, or pathogen exposure. They elicit a conserved homeostatic cell danger response [172] that could result in the cell shifting to an alternative resting steady state and contribute to ME/CFS pathology [34]. An alternative explanation would involve chronic ongoing exposure of cells to a causative insult. However, chronic pathogen exposure as the precipitating factor for such a response in ME/CFS seems unlikely, as theories of viral persistence have been thoroughly investigated and the evidence for chronic viral infection in ME/CFS is inconsistent [173]. 

Another feature of ME/CFS is the characteristic symptom heterogeneity. Patient histories suggest that the initial insult eliciting the shift to the ME/CFS state differs between individuals and so may dysregulate the underlying cellular stress signaling pathways in different ways. This could contribute to inconsistencies between various studies and highlights the need for sensitive diagnostics and patient stratification into recognizably different categories. 

No matter the initial insult, each of the aforementioned possibilities can involve the interaction between chronic inflammation and immunological and mitochondrial dysfunction [99,174,175]. This is similar to theories previously presented by others pertaining to dysregulated homeostatic feedback loops [34]. Faced with a new challenge, such as the re-emergence of a dormant pathogen, over-exertion, or other stress depending on the tolerance of the individual, the affected pathways could again be perturbed, triggering a molecular cascade during bouts of PEM. In some patients, this may mimic the response to a recurrent infection regardless of whether the new trigger is pathogen-driven. Symptom flare-ups characteristic of ME/CFS may therefore result from antagonization of just one of the underlying nodes of a multi-system pathological web and a symptomatic body-wide cascade ensues. 

For example, the elevated oxidative stress reported in the disorder [148,149,150,151,152,153] may be entangled with perturbed immune-inflammatory pathways [175,176,177,178], gut inflammation [158], and dysfunctional mitochondria. This may be exacerbated by other disturbances, such as the reported reduction in CK levels [15], which can lead to the absence of CK-mediated ROS suppression [179]. This raises the possibility of a vicious cycle of immunodysregulation and gut dysbiosis accompanied by poor physiological gut function [122] that contribute to the perpetuation of a chronic bowel disease state. Such an altered state could interact with the previously suggested HPA dysregulation [34] and contribute to the perpetuation of an alternative resting homeostasis.

### 5.2. Identifying Underlying Mechanisms

The earlier sections of this review have discussed the evidence for dysregulated biological mechanisms in ME/CFS patients at the molecular, cellular, tissue, organ, and whole body levels. These changes are summarized in Table 1 and together provide insights into potential underlying disease mechanisms.

Based on current biomedical literature, perturbations to the various systems listed in Table 1 are implicated in the underlying pathological mechanisms in ME/CFS. These phenomena are correlated clinically in that they appear in people with the disorder. However, the causal links between them are unknown and cannot be discerned purely on the basis of clinical correlation. There are many scenarios where more than one of the affected systems may exert pathological effects on another and vice versa. This complex and often reciprocal regulatory cross-talk between systems makes it difficult to distinguish cause from effect, so there is great need for the potential causal relationships to be addressed directly using appropriate experimental models. 

For example, the potential impact of gut hyperpermeability on body-wide mitochondrial function has been outlined in Section 4.2. However, the reverse may also take place. The differentiation, proliferation, and function of intestinal epithelial cells is known to depend upon normal mitochondrial function, with mitochondrial dysfunction resulting in subsequent hyperpermeability of the intestinal wall [188,189,190]. Therefore, it is impossible to distinguish cause from effect in this case with correlations alone. Similarly, changes in metabolite levels in ME/CFS have been associated with both IBS comorbidity [31] and alterations to intestinal flora composition [118]. However, it has not been experimentally shown whether the IBS is caused by host metabolic dysfunction or irregular metabolite production and excretion by an altered gut microbiota, or if the gut microflora and metabolic abnormalities are caused by the IBS.

The influence of the mitochondria upon the function of the immune system has similar implications. The mitochondria can partake in activation or suppression of inflammation indirectly by regulating autophagy, which in turn bears downstream regulatory consequences for the activation of inflammation [191]. Mitochondria also regulate elements of the innate immune system by the release of immunogenic ligands [178] and are important for immune cell effector function due to their classical roles in energy production and metabolism. Furthermore, immunological dysfunction is common in conditions which are primarily mitochondrial diseases [192]. This is an important consideration with regard to the gut microbiota since it, in turn, influences and is influenced by the function of the immune system and [128,163,193,194] immune cell population composition [195]. Therefore, changes to any one of the mitochondria, immune system, or gut may dysregulate the others, again emphasizing the importance of future research addressing cause–effect relationships. 

## 6. Conclusions

ME/CFS is a heterogeneous condition that may encompass scenarios where uncertain, and possibly varying, underlying insults trigger body-wide molecular and cellular perturbations perpetuated by an alternative stable homeostatic states. Diagnostic advancement and the development of tools which objectively and accurately phenotype patients is therefore paramount for the development of mechanistic insight and effective therapeutics. 

It is likely that the inflammation and immune dysfunction classically studied in ME/CFS are entangled with dysfunctional energetics, gut health, or autonomic and adrenal dysregulation. The evidence for metabolic and mitochondrial dysfunction indicates inefficient respiration, impaired provision of TCA cycle substrate, and metabolic shifts towards the utilization of alternative metabolites. Immune effector cell dysfunction, chronic inflammation, defective signaling, and elevated oxidative stress may interact with not only the dysfunctional energetics but also with abnormal gut physiology and microbiota composition. These effects on the gut may also tie back to mitochondrial function and vice versa. The reciprocal interactions between these affected systems and the varied clinical presentation of relevant symptoms between individuals make it difficult to postulate cause–effect relationships with confidence. Furthermore, while disturbances to this range of interconnected systems across the body have been demonstrated, in some cases concurrently, this body of research has historically relied upon correlations, which creates the urgent need for research utilizing direct experimental investigation of cause–effect relationships.

## Figures and Tables

**Table 1:** Brief summary of relevant reports contributing towards the biomedical basis of ME/CFS.

Area of Study	Brief Summary of Key Reports in ME/CFS
Metabolomics	Multiple reports of disturbed amino acid metabolism [6,38,39,40,180]. Dysregulated lipid metabolism [7,13,31,39], possible glycolysis impairment [39], possible PDH impairment [40], urea cycle dysregulation [6,38]. Overall: TCA cycle substrate provision deficiency and reliance on alternative fuel sources.
Mitochondrial function	Reduced [9] vs. elevated steady-state ATP levels [42,44,48] but resting ATP synthesis rates are normal [41,45]. Complex V inefficient and compensated for by upregulation of supporting pathways [41].
Muscle activity	Unresponsive AMPK and reduced glucose [43,48,141] and oxygen uptake [181].
Natural killer cells	Overall inconsistent evidence—role mostly unknown [65,66,67,68,69,70,71,72].
Calcium signaling	Evidence for impaired TRPM3 function [81,82].
Inflammation and cytokines	Inconsistent molecular evidence [102,182], yet is likely to play a role based on clinical presentation and the many reported disturbances in related systems.
Autoimmunity	Little researched, but proposed to form a subtype comorbid with IBS [114]. Role for B cell-mediated autoimmunity challenged by negative outcome of rituximab trial [106,109,110].
B cells	Linked to mitochondrial disturbances, subset proportions may vary [41,45,183].
Gut microbiota and physiology	Widely reported disturbances to the gut microbiota [12,103,115,116,117,119,120,184] and gut hyperpermeability [103,123,124].
Autonomic and hormonal dysregulation	Vasomotor abnormalities [165,185], hypocortisolism [169,170], broad HPA axis disturbance [186,187].
